# Association of egg intake with risks of cardiometabolic factors among adults in China

**DOI:** 10.3389/fpubh.2022.1010539

**Published:** 2022-10-26

**Authors:** Yingying Jiao, Weiyi Li, Hongru Jiang, Liusen Wang, Shaoshunzi Wang, Lixin Hao, Xiaofang Jia, Zhihong Wang, Huijun Wang, Bing Zhang, Gangqiang Ding

**Affiliations:** ^1^National Institute for Nutrition and Health, Chinese Center for Diseases Control and Prevention, Beijing, China; ^2^Key Laboratory of Trace Elements and Nutrition, National Health Commission, Beijing, China; ^3^Dietary Reference Intakes (DRIs) Expert Committee on Macroelements, Chinese Nutrition Society, Beijing, China

**Keywords:** adults, egg intake, cardiometabolic risk factors, dose-response relationship, China

## Abstract

**Objective:**

To explore the association between egg intake and cardiometabolic factors (CMFs) in Chinese adults.

**Method:**

The subjects were 6,182 adults aged 18–64 who had complete survey data and had no CMFs at baseline. Egg intake was assessed with 3 days−24 h dietary recalls in all waves of the China Health and Nutrition Survey (CHNS). Multivariate Cox proportional risk regression model and restricted cubic spline (RCS) model were used to analyze the association and dose-response relationship between egg intake and CMFs.

**Results:**

Of the 6,182 participants who did not have metabolic syndrome (MetS) at baseline, 1,921 developed this disease during an average follow-up of 5.71 years, with an incidence of 31.07%. Central obesity, elevated TG, decreased HDL-C, elevated blood pressure and elevated plasma glucose were 38.65, 26.74, 30.21, 40.64, and 30.64%, respectively. After adjusting for demographic characteristics, lifestyle, energy and BMI, using the lowest quintile (Q1) as a reference, the risk of central obesity, elevated TG, decreased HDL-C, and elevated plasma glucose in the highest quintile (Q5) were reduced by 15% (HR = 0.85, 95% CI = 0.73–0.98, *P* = 0.16), 33% (HR = 0.67, 95% CI = 0.57–0.78), 25% (HR = 0.75, 95% CI = 0.63 0.90, *p* = 0.05), and 28% (HR = 0.72, 95% CI = 0.63–0.83, *p* < 0.05), respectively. The risk of elevated blood pressure was reduced by 26% in the fourth quintile (HR = 0.74, 95% CI = 0.64–0.85, *P* = 0.85). RCS analysis show that the overall correlation and nonlinear relationship between egg intake and CMFs were statistically significant (*P* < 0.05). When the intake was lower than 20 g/days, the risk of MetS, central obesity, elevated blood pressure and elevated plasma glucose were negatively correlated with egg intake, while elevated TG was negatively correlated with eggs when the intake was lower than 60 g/days. There was no statistically significant association between egg intake and CMFs at higher egg intake.

**Conclusion:**

There was a *U*-shaped association between egg intake and CMFs in Chinese adults.

## Introduction

Cardiovascular disease (CVD) is the main cause of death and disability all over the world (1), ranking first among the causes of death of urban and rural residents in China and 46. 66% in rural and 43.81% in urban. Two out of every five deaths are due to CVD (2). Among many risk factors, cardiometabolic factors (CMFs), including obesity, elevated blood pressure, elevated plasma glucose and dyslipidemia, are the main risk factors of CVD, which are also the important components of the “health factors” in the “Life's Essential 8” newly proposed by the American Heart Association (3). The prevalence of each CMF increased by 20–50% from 2009 to 2018 in China (4). Studies have found that more than half of people had at least two CMFs in China (5), and the prevalence of metabolic syndrome (MetS) is increasing at home and abroad, and the data from China Health and Nutrition Survey (CHNS) in 2015 showed that the prevalence reached 18.1% (6). It places a heavy burden on individuals and societies.

Eggs are the main source of dietary cholesterol, containing about 71% of the recommended daily intake of cholesterol (7). Because of its affordable market price, it is widely consumed worldwide. Evidence for the relationship between egg consumption and human health is controversial, with some studies finding a reduction in the risk of hemorrhagic stroke and levels of inflammatory factors, but no association in others (e.g., cancer, CVD, etc.) (8, 9). Eggs were found to be negatively associated with MetS in a prospective study in Korea and a cross-sectional study in China (7, 10), while a positive association was found in an Iranian cohort (11), and no significant association was found in an Australian and another Korean cohort (12, 13). At present, there are limited studies on eggs and CMFs at home and abroad, and most of them are cross-sectional studies. Therefore, this study uses the follow-up data from CHNS in 2009, 2015, and 2018 to analyze the association between egg intake and CMFs, and provide scientific basis for effective prevention and control of related diseases.

## Materials and methods

### Study population

We used data from CHNS, a long-term longitudinal follow-up project jointly conducted by the Institute of Nutrition and Health, Chinese Center for Disease Control and Prevention and the University of North Carolina at Chapel Hill. The project was launched in 1989 and has completed 11 waves of follow-up. And it was conducted in 15 provinces, the specific provinces or cities are shown in Supplementary Table 1. A stratified multistage random cluster sampling method was used, with county neighborhood committee, urban neighborhood committee, village and suburban village as the basic survey points. Twenty households were randomly selected from each survey point, and all household members were investigated. The same households and household members were tracked as far as possible in each round of survey. The survey content included questionnaire survey (community, household and personal information), medical physical examination (blood biochemical test was added in 2009, 2015, and 2018) and dietary survey. For specific sampling methods, survey scheme and content, please refer to the literature (14–16).

**Figure 1:**
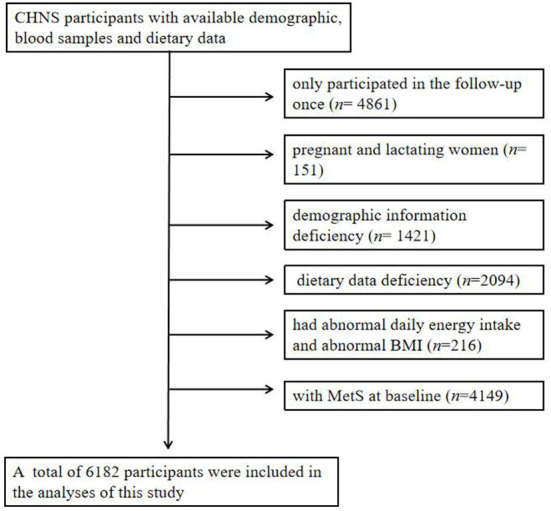
Flowchart of subjects.

In this study, we selected adults aged 18–64 as subjects, who participated in at least two follow-up surveys in 2009, 2015, and 2018. We excluded pregnant and lactating women (*n* = 151), those having demographic information deficiency (*n* = 1,421), having dietary data deficiency (*n* = 2,094), having abnormal daily energy intake (man: >6,000 or < 800 kcal; women: 4,000 or < 600 kcal) (17) and abnormal body mass index (BMI) (< 14.0 or > 45.0 kg/m^2^) (*n* = 216) (5) and those having MetS at baseline (*n* = 4,149). Finally, 6,182 subjects were included in this study (Figure 1). In addition, we identified specific study population subgroups for each single CMFs. The project was reviewed by the Ethics Review Committee of Institute of Nutrition and Health of Chinese Center for Disease Control and Prevention (No. 201524), and all the participants signed the informed consent.

### Dietary assessment

In each wave of surveys, the consumption of food data was collected by the consecutive 3 days−24 h dietary recalls, cooking oil and condiments was collected by household weighing method and distributes them to individuals according to the individual energy consumption ratio in the household. Then, Food Composition Table was used to convert the collected consumption of various foods, edible oils and condiments into the intake of energy and nutrients. Egg and energy intake were included in this study. In prospective analyses, considering that potential changes in diet after the development of the disease may confound the relationship between egg intake and CMFs, updating of dietary information was stopped upon diagnosis of the disease. If the subjects entered the cohort in 2009 and developed the disease in 2015, the egg consumption in 2009 was used; if the subjects developed the disease in 2018, the average egg consumption in 2009 and 2015 was used. If the subjects entered the cohort in 2015 and developed disease in 2018, the egg consumption in 2015 was used.

### Diagnostic criteria of CMFs

We defined CMFs according to the joint statement of the International Diabetes Federation (IDF) in 2009 (18), Central obesity: waist circumference ≥85 cm in men and ≥80 cm in women; Elevated triglyceride (TG): ≥1.7 mmol/L or under treatment; Decreased HDL-C: < 1.0 mmol/L in men and < 1.3 mmol/L in women or under treatment; Elevated blood pressure: systolic blood pressure ≥130 mmHg, diastolic blood pressure ≥85 mmHg or being treated for essential hypertension; Elevated plasma glucose: fasting plasma glucose (FPG) ≥5.6 mmol/L or previously diagnosed with diabetes. The presence of any 3 of 5 risk factors constitutes a diagnosis of MetS. Detailed description of measurement methods was provided in this literature (19).

### Covariates

The demographic data, lifestyle, dietary information were obtained through face-to-face surveys with special questionnaires by uniformly trained and qualified investigators. Such as age (18–49 and 50–64 years), gender, annual household income, education (low: primary school and below; medium: middle or high school; high: college and above), residential area (urban or rural), smoking (yes or no), drinking habit (yes or no), physical activity, energy and BMI (< 18.5, 18.5~23.9, and ≥24.0 kg/m^2^). Annual household income and energy were categorized into three groups according to the tertiles (low, medium, and high). Physical activity was assessed by the metabolic equivalent (MET) and duration of each activity (hours/week) (20, 21) and was divided into three groups according to the tertiles. Energy was adjusted according to the tertiles. BMI is calculated by height and weight measurements.

### Statistical analysis

Continuous and categorical variables were described by mean ± standard deviation and percentage (%), respectively. Chi-square test and ANOVA were used to analyze the baseline characteristics of the subjects according to the quintile of egg intake. The association between egg intake and CMFs was analyzed by multivariate Cox proportional risk model across quintile groups of egg intake, and we calculated *p*-values for linear trend by using the median value for each group of egg intake. We calculated hazard ratios (HRs; 95% CIs) and constructed three sequential models including demographic characteristics, lifestyle, energy and BMI. Finally, RCS model with 5 knots was used to analyze the dose-response relationship between egg intake and CMFs. All data were analyzed by SAS (version 9.4, SAS Institute, Inc., Cary, NC, USA) and R software version 4.1.0 (The R Foundation for Statistical Computing), and we defined statistical significance as *p* < 0.05.

## Results

### Baseline characteristics

As presented in Table 1, using the lowest quintile (Q1) as a reference, people with higher egg intake were more likely to be male, with higher income and education, and had lower physical activity, higher energy intake, higher BMI and had a drink habit. Baseline waist circumference, systolic blood pressure, diastolic blood pressure, and fasting plasma glucose were different between groups (*p* < 0.05), other variables including age, BMI, smoking, and baseline TG and HDL-C levels were not significantly different between egg intake levels (*p* > 0.05).

**Table 1:** Characteristics of participants according to quintile of egg intake.

	**Egg intake (g/day)**					***p*-value**
	**Q1**	**Q2**	**Q3**	**Q4**	**Q5**	
	**(0.00)**	**(0.00~14.67)**	**(14.67~29.63)**	**(29.63~50.00)**	**(50.00~)**	
Age, %						0.275
18~49	59.48	57.21	61.34	61.72	59.27	
50~64	40.52	42.79	38.66	38.28	40.73	
Male,%	45.40	42.21	43.07	44.59	50.00	0.002
Household income per capita,%						< 0.001
Low	39.30	30.29	30.51	32.95	29.47	
Median	33.65	32.94	33.61	33.28	32.91	
High	27.05	36.76	35.89	33.77	37.62	
Education,%						< 0.001
Primary and below	38.86	31.18	28.87	29.67	27.00	
Middle and high	47.01	48.82	51.55	49.10	50.48	
College and above	14.14	20.00	19.58	21.23	22.52	
Urban,%	31.49	34.56	34.50	36.23	34.19	0.091
Never smoked,%	68.74	71.32	72.19	71.89	71.09	0.223
Never drunk alcohol,%	68.51	70.15	69.25	67.38	63.90	0.017
Physical activity,%						< 0.001
Low	29.21	35.88	34.09	32.62	37.78	
Median	31.93	33.24	32.30	36.07	33.79	
High	38.86	30.88	33.61	31.31	28.43	
BMI (kg/m^2^),%						< 0.001
< 18.5	7.10	7.94	6.69	5.57	3.67	
18.5~23.9	61.09	65.74	60.03	58.93	58.31	
≥24.0	31.82	26.32	33.28	35.49	38.02	
Energy (kcal/d)						< 0.001
Low	35.48	39.85	36.30	30.33	26.68	
Median	30.60	31.76	34.75	37.05	33.15	
High	33.92	28.38	28.96	32.62	40.18	
WC (cm)	79.32 ± 9.85	78.88 ± 9.21	79.61 ± 0.49	80.21 ± 0.29	81.62 ± 10.48	< 0.001
TG (mmol/l)	1.25 ± 0.88	1.22 ± 0.96	1.22 ± 0.78	1.21 ± 0.80	1.19 ± 0.72	0.935
HDL-C (mmol/l)	1.45 ± 0.35	1.45 ± 0.34	1.46 ± 0.35	1.50 ± 0.53	1.48 ± 0.40	0.457
SBP (mmHg)	118.84 ± 14.95	118.70 ± 15.59	119.01 ± 15.07	119.29 ± 14.56	120.20 ± 15.01	0.009
DBP (mmHg)	77.35 ± 9.64	76.95 ± 9.57	77.66 ± 9.83	77.78 ± 9.94	78.71 ± 9.75	< 0.001
FPG (mmol/l)	5.05 ± 0.97	5.00 ± 0.94	4.98 ± 0.73	5.06 ± 0.97	5.11 ± 0.85	< 0.001

WC, waist circumference; TG, triglyceride; HDL-C, high density lipoprotein cholesterol; SBP, systolic blood pressure; DBP, diastolic blood pressure; FPG, fasting plasma glucose.

### Association of egg intake with CMFs

Among the 6,182 participants who did not have MetS at baseline, 1,921 developed during an average follow-up of 5.71 years, with an incidence of 31.07%. After adjusting for demographic characteristics, lifestyle, energy, and BMI, the risk of MetS was reduced by 15% (HR = 0.85, 95% CI = 0.74–0.97) in the highest quintile group (Q5) using the lowest quintile group (Q1) as a reference. The trend test had no statistical significance (*p* = 0.12; Table 2).

**Table 2:** Hazard ratio (HR) and 95% confidence interval (CI) for association of egg intake with CMFs.

	**Egg intake (g/day)**					***P* trend**
	**Q1**	**Q2**	**Q3**	**Q4**	**Q5**	
**MetS** ^**a**^						
Median	0.00	10.00	20.00	37.05	66.66	
Model1	1.00	0.62 (0.52, 0.74)*	0.81 (0.71, 0.93)*	0.78 (0.68, 0.89)*	0.92 (0.81, 1.04)	0.953
Model2	1.00	0.61 (0.51, 0.73)*	0.82 (0.71, 0.94)*	0.78 (0.68, 0.89)*	0.90 (0.79, 1.02)	0.796
Model3	1.00	0.64 (0.53, 0.78)*	0.76 (0.66.0.88)*	0.71 (0.62, 0.83)*	0.85 (0.74, 0.97)*	0.119
**Central obesity** ^**b**^						
Median	0.00	8.00	20.00	35.14	65.88	
Model1	1.00	0.56 (0.46, 0.68)*	0.82 (0.71, 0.95)*	0.73 (0.63, 0.84)*	0.87 (0.76, 1.00)	0.394
Model2	1.00	0.56 (0.45, 0.68)*	0.82 (0.71, 0.95)*	0.71 (0.61, 0.83)*	0.86 (0.74, 0.99)*	0.255
Model3	1.00	0.59 (0.47, 0.73)*	0.80 (0.69, 0.93)*	0.70 (0.60, 0.82)*	0.85 (0.73, 0.98)*	0.158
**Elevated TG** ^**c**^						
Median	0.00	10.00	20.00	36.67	65.60	
Model1	1.00	0.77 (0.64, 0.91)*	0.75 (0.65, 0.88)*	0.65 (0.56, 0.76)*	0.68 (0.59, 0.79)*	< 0.001
Model2	1.00	0.76 (0.64, 0.90)*	0.76 (0.65, 0.88)*	0.65 (0.55, 0.75)*	0.67 (0.58, 0.78)*	< 0.001
Model3	1.00	0.75 (0.63, 0.91)*	0.75 (0.64, 0.88)*	0.63 (0.53, 0.74)*	0.67 (0.57, 0.78)*	< 0.001
**Decreased HDL-C** ^**d**^						
Median	0.00	10.00	21.82	40.00	70.00	
Model1	1.00	0.53 (0.42, 0.66)*	0.74 (0.63, 0.88)*	0.72 (0.60, 0.85)*	0.76 (0.64, 0.90)*	0.044
Model2	1.00	0.51 (0.40, 0.64)*	0.73 (0.61, 0.86)*	0.69 (0.58, 0.83)*	0.75 (0.63, 0.89)*	0.040
Model3	1.00	0.50 (0.39, 0.63)*	0.70 (0.59, 0.84)*	0.67 (0.56, 0.81)*	0.75 (0.63, 0.90)*	0.055
**Elevated BP** ^**e**^						
Median	0.00	10.00	20.00	36.67	66.08	
Model1	1.00	0.59 (0.49, 0.71)*	0.84 (0.74, 0.96)*	0.79 (0.70, 0.91)*	0.92 (0.82, 1.05)	0.877
Model2	1.00	0.58 (0.48, 0.70)*	0.84 (0.74, 0.97)*	0.79 (0.69, 0.90)*	0.92 (0.81, 1.05)	0.852
Model3	1.00	0.55 (0.46, 0.67)*	0.80 (0.69, 0.91)*	0.74 (0.64, 0.85)*	0.92 (0.80, 1.05)	0.850
**Elevated FPG** ^**f**^						
Median	0.00	10.00	20.00	36.27	65.38	
Model1	1.00	0.52 (0.43, 0.62)*	0.73 (0.64, 0.84)*	0.68 (0.59, 0.78)*	0.74 (0.64, 0.84)*	0.003
Model2	1.00	0.51 (0.42, 0.61)*	0.75 (0.65, 0.86)*	0.69 (0.59, 0.79)*	0.74 (0.64, 0.84)*	0.004
Model3	1.00	0.50 (0.41, 0.61)*	0.72 (0.62, 0.83)*	0.66 (0.57, 0.76)*	0.72 (0.63, 0.83)*	0.002

^*a*^N = 6,182. ^*b*^N = 4,075. ^*c*^N = 5,520. ^*d*^N = 3,774. ^*e*^N = 4,718. ^*f*^N = 5,611. Model 1 adjusted age, sex, education, urban and rural areas, and income; In model 2, smoking, drinking and physical activity levels were further adjusted based on Model 1. In Model 3, energy intake and BMI were further adjusted based on Model 2.

*P < 0.05.

Analysis of CMFs found that the incidence of central obesity, elevated TG, decreased HDL-C, elevated blood pressure and elevated plasma glucose were 38.65, 26.74, 30.21, 40.64, and 30.64%, respectively. After adjusting for all the covariables, taking Q1 as the reference group, the risk of central obesity, elevated TG, decreased HDL-C and elevated plasma glucose were reduced by 15 (HR = 0.85, 95% CI = 0.73–0.98), 33 (HR = 0.67, 95% CI = 0.57–0.78), 25 (HR = 0.75, 95% CI = 0.63–0.90) and 28% (HR = 0.72, 95% CI = 0.63–0.83) in the highest intake (Q5), respectively. Elevated blood pressure was associated with a 26% lower risk (HR = 0.74, 95% CI = 0.64–0.85) in the Q4 group, and there was no statistically significant association in the Q5 group. The trend test was statistically significant only for elevated TG and elevated plasma glucose (*p* < 0.05; Table 2).

### Dose-response relationship between egg intake with CMFs

**Figure 2:**
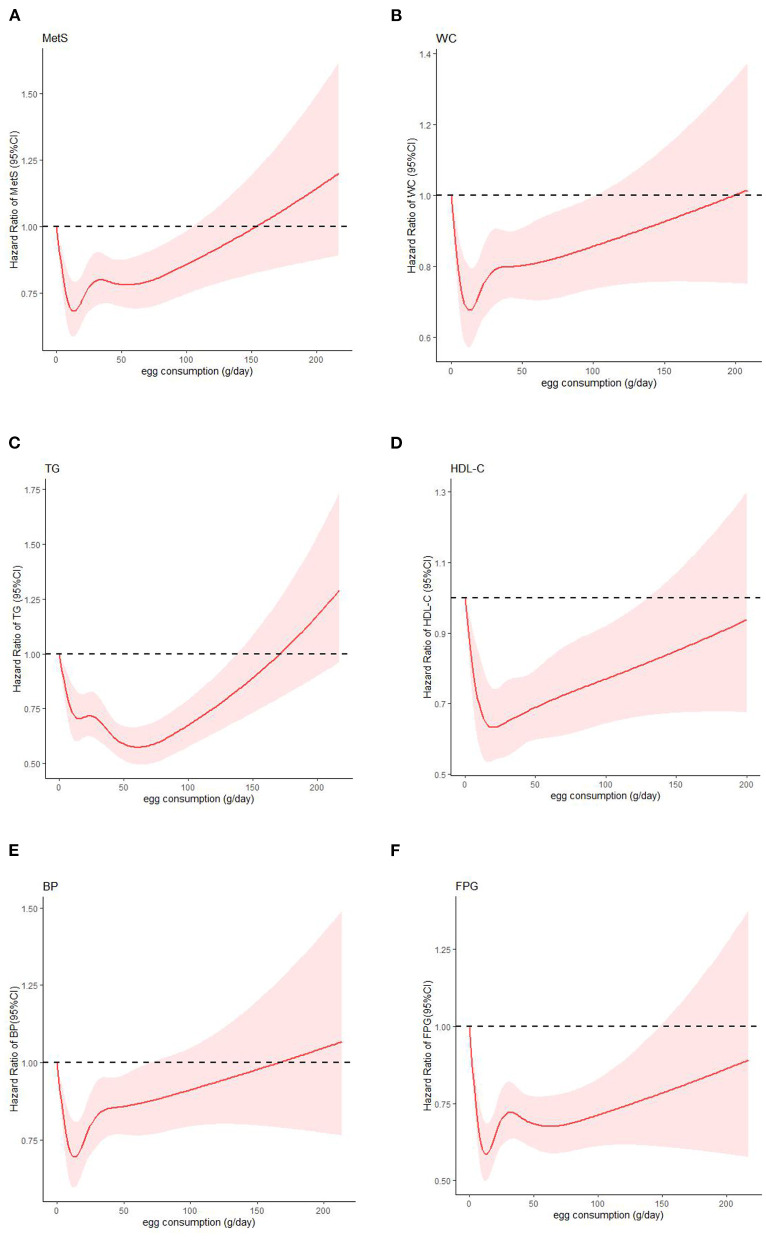
Dose-response relationship of egg intake with CMFs: **(A)** MetS: metabolic syndrome; **(B)** WC: waist circumference; **(C)** TG: triglyceride; **(D)** HDL-C: high density lipoprotein cholesterol; **(E)** BP: blood pressure; **(F)** FPG: fasting plasma glucose.

RCS analysis showed a *U*-shaped association between egg intake and CMFs. Taking no egg intake as reference, when the egg intake was < 20 g/days, the risk of CMFs except TG decreased significantly with the increase of egg intake. When the intake was >20 g/days, the risk increased, but egg intake still had a protective effect (HR < 1). When the intake was >100 g/days, egg intake was not statistically associated with MetS and central obesity, and there was no statistical association with decreased HDL-C, elevated blood pressure and elevated plasma glucose when the intake was >150, 75, and 150 g/days, respectively. In addition, Egg intake was negatively correlated with elevated TG when the intake was < 60 g/days, and no longer had protective effect after 150 g/days (Figure 2).

## Discussion

The study analyzed the association and dose-response relationship between egg intake and CMFs in adults aged 18–64 years in 15 provinces of China and showed a *U*-shaped association between them. Consistent results were found in a cross-sectional study of 23,993 Korean adults aged 19 and above from the Korea National Health and Nutrition Examination Survey (KNHANES) 2007–2011, but dietary survey was conducted using a food frequency method in this study (22).

### Association between eggs and MetS and its mechanism

A prospective study of 1,633 Koreans showed that compared with those who consumed no eggs a week, those who consumed >3 eggs per week had a 54% (RR = 0.46, 0.26–0.82) and 46% (RR = 0.54, 0.31–0.93) lower risk of MetS in men and women, respectively (7). In a study of 3,616 iranians, the risk of egg consumption was 2.7 times than that of non-consumption (11). In the two cohorts of 5,251 Koreans and 5,324 Australians, compared with the lowest quartile group, no significant association was found between egg intake and MetS in the highest quartile group (12, 13). In a cross-sectional study of 11,529 people in China, it was found that the risk of MetS in people who consumed >7 eggs per week was 18% lower than that those who consumed < 3 eggs (OR = 0.82, 95% CI = 0.74–0.91) (10). In another cross-sectional study of 8,241 people in China, it was found that the risk of MetS of consuming >1 egg per day was 1.18 times that of consuming < 1/2 egg per day (40–50 g per egg) (23).

The results are controversial between egg consumption and MetS, which may have the following reasons. 1) There may be an effect of racial differences; 2) some studies have found that the association may be primarily driven by egg consumption patterns (7, 24). For example, In the United States, egg consumption reflects adherence to Western dietary patterns, as eggs are often eaten with red or processed meat, refined grains, and sugary beverages. Even careful adjustment of foods commonly consumed with eggs will not completely eliminate residual confounding associated with egg consumption habits; 3) In addition, the length of follow-up in the cohort study may also be related to the inability to draw conclusions about the long-term effects of egg intake; 4) Differences in the covariates adjusted for in the study may have influenced the results.

It has been found that the inverse association between egg intake and MetS may be attributed to other components of eggs rather than cholesterol, such as lutein and zeaxanthin, which may influence the development of MetS by improving lipoprotein metabolism and plasma carotenoid status (25). The explanation for the positive correlation was mainly attributed to cholesterol (23). In addition, eggs are rich in choline, and the plasma trimethylamine-N-oxide produced by intestinal microbiota metabolism may contribute to the positive correlation between egg intake and MetS (26). Studies have shown that the nutritional benefits of an egg far outweigh the adverse effects of the cholesterol it contains. The Dietary Guidelines for Americans (2020–2025) recommend eggs as a part of a healthy diet and remove the daily dietary cholesterol limit of 300 mg. The dietary Guidelines for Chinese Residents in 2022 put forward the recommended intake of eggs of 280~350 g per week, but the population within the recommended range only accounts for 13.9% (27). Therefore, it is suggested to strengthen the propaganda and education on the nutritional value and health effects of eggs, improve the health awareness of residents, and increase the egg intake of Chinese residents in an appropriate amount.

### Association between eggs and five CMFs and its mechanism

Our study found that egg intake reduced the risk of CMFs within a range. Similar results were found in a cross-sectional study of 23,993 subjects in Korea (22). In South Korea's Yangpyeong Cohort (*n* = 3,616), the study found that compared with no consumption of eggs, consuming >3 eggs per week was negatively associated with elevated TG and elevated plasma glucose only in men (7). Another large-scale genomic community-based study conducted in Korea (*n* = 130,420) found that compared with < 1 egg per week, higher egg intake (≥7 eggs per week) was associated with lower risk of five CMFs in women and lower risk of decreased HDL-C in men (28). This indicates that gender may also affect the association between egg intake and disease in addition to racial differences. In our study, gender interaction was conducted on the sample population in advance and no statistical difference was found (*p* = 0.14), so there was no stratified analysis of gender.

Eggs serve as a source of high-quality protein, some studies have reported that egg intake can increase satiety and reduce calorie intake (29, 30), and it has also been shown to promote weight loss in limited human studies (31, 32), which may be related to the reduced incidence of central obesity. Another intervention study found that eggs decreased small LDL particles (33), which were highly correlated with decreased HDL-C and elevated TG (34). The negative correlation between eggs and elevated plasma glucose can be explained by the egg-induced decrease in inflammation (35), which may be due to the increased insulin sensitivity from monounsaturated fatty acids, polyunsaturated fatty acids, and antioxidants (lutein, zeaxanthin, and folic acid) in eggs (7). However, a positive association was found between eggs and diabetes, mainly in Americans, which was believed to be driven by egg consumption patterns. In addition, choline metabolites may be related to the relationship between egg intake and diabetes mellitus (24). Finally, omega-3 fatty acids high in eggs can compete with arachidonic acid in the cyclooxygenase pathway to decrease blood pressure (7).

Our study provided prospective evidence of non-linear association between egg intake and CM risk factors. In addition, for the classification of continuous variable, the number and boundary value of the classification are often subjective, which may lead to bias of research results (36). Therefore, the dose-response relationship between egg intake and CMFs was further analyzed by RCS model, and the results were consistent. However, there are still some limitations: 1) The 3 days−24 h dietary recalls has the recall bias, and it usually cannot evaluate daily dietary intake. To investigate long-term dietary behavior, it is better to use the food frequency questionnaire (FFQ) to collect dietary information, but in calculating nutrients, the 3 days−24 h dietary recalls is more accurate than FFQ; 2) We cannot rule out the possible influence of healthy dietary patterns associated with egg consumption; 3) Menopausal status, hormone use and oral contraceptives were not examined in this study, which may have influenced the results; 4) The different cooking methods of eggs may have influenced the results; 5) The influence of unknown confounding factors may exist; 6) We found that most dietary surveys abroad use FFQ method, while China mostly uses 3 days−24 h dietary recalls, which should be cautious when comparing and extrapolate the results.

## Conclusion

In summary, our results suggest an overall *U*-shaped association between egg intake and CMFs. More prospective studies are needed to verify the differences in association and the possible mechanisms among different CMFs in future.

## Data availability statement

The original contributions presented in the study are included in the article/Supplementary material, further inquiries can be directed to the corresponding author/s.

## Ethics statement

The studies involving human participants were reviewed and approved by the Ethics Review Committee of Institute of Nutrition and Health of Chinese Center for Disease Control and Prevention (No. 201524). The patients/participants provided their written informed consent to participate in this study.

## Author contributions

YJ data collation, statistical analysis, and paper writing. WL, HJ, LW, SW, LH, and XJ data collection, paper revision, and guidance. HW and BZ research guidance, paper review, and administrative support. ZW and GD research design, funding support, paper revision, and review. All authors have read and agreed to the published version of the manuscript.

## Funding

This work was funded by National Key R&D Program of Ministry of Science and Technology-Research Project on Dietary Nutrition Assessment and Intervention Techniques (No. 2020YFC2006300), International Cooperation Project [China Health and Nutrition Survey (Nos. R01-HD30880, DK056350, R24 HD050924, and R01-HD38700)], and National Financial Project [Operation of Public Health Emergency Response Mechanism (No. 131031107000210002)].

## Conflict of interest

The authors declare that the research was conducted in the absence of any commercial or financial relationships that could be construed as a potential conflict of interest.

## Publisher's note

All claims expressed in this article are solely those of the authors and do not necessarily represent those of their affiliated organizations, or those of the publisher, the editors and the reviewers. Any product that may be evaluated in this article, or claim that may be made by its manufacturer, is not guaranteed or endorsed by the publisher.
