# Supplementary material for: Association of egg intake with risks of cardiometabolic factors among adults in China
Source: Front Public Health. 2022 Oct 26;10:1010539. doi: 10.3389/fpubh.2022.1010539 (PMC9645429; doi:10.3389/fpubh.2022.1010539)
Supplement: Supplementary file 1 [file Table_1.docx]

**Figure 1.**


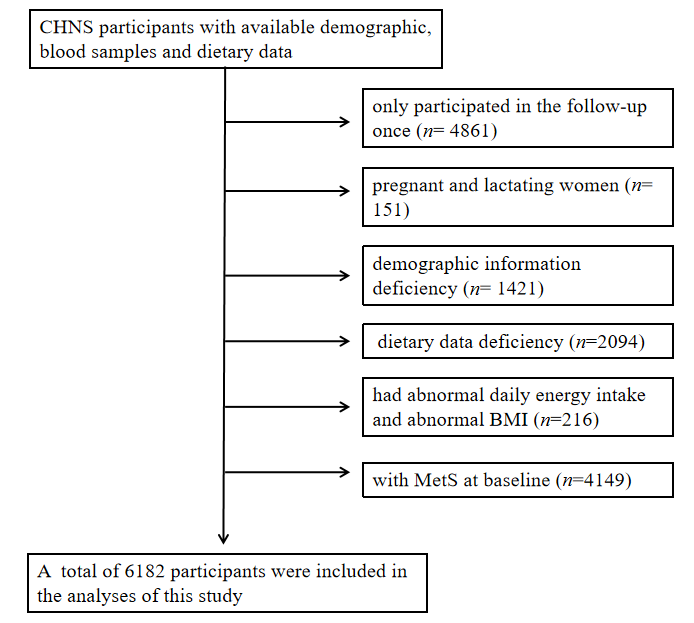


**Supplementary Table 1.**

Supplementary Table 1 Provinces, municipalities and numbers surveyed in different years

| year | Provinces or Cities | Total Number |
| --- | --- | --- |
| 1989 | Shandong, Henan, Guangxi, Liaoning, Jiangsu, Hubei, Hunan, Guizhou (8) | 15915 |
| 1991 | Shandong, Henan, Guangxi, Liaoning, Jiangsu, Hubei, Hunan, Guizhou (8) | 14739 |
| 1993 | Shandong, Henan, Guangxi, Liaoning, Jiangsu, Hubei, Hunan, Guizhou (8) | 13872 |
| 1997 | Shandong, Henan, Guangxi, Jiangsu, Hubei, Hunan, Guizhou, Heilongjiang (8) | 14422 |
| 2000 | Shandong, Henan, Guangxi, Liaoning, Jiangsu, Hubei, Hunan, Guizhou, Heilongjiang (9) | 15696 |
| 2004 | Shandong, Henan, Guangxi, Liaoning, Jiangsu, Hubei, Hunan, Guizhou, Heilongjiang (9) | 12389 |
| 2006 | Shandong, Henan, Guangxi, Liaoning, Jiangsu, Hubei, Hunan, Guizhou, Heilongjiang (9) | 11895 |
| 2009 | Shandong, Henan, Guangxi, Liaoning, Jiangsu, Hubei, Hunan, Guizhou, Heilongjiang (9) | 11979 |
| 2011 | Shandong, Henan, Guangxi, Liaoning, Jiangsu, Hubei, Hunan, Guizhou, Heilongjiang, Beijing, Shanghai, Chongqing (12) | 15510 |
| 2015 | Shandong, Henan, Guangxi, Liaoning, Jiangsu, Hubei, Hunan, Guizhou, Heilongjiang, Beijing, Shanghai, Chongqing, Shaanxi, Yunnan and Zhejiang (15) | 20230 |
| 2018 | Shandong, Henan, Guangxi, Liaoning, Jiangsu, Hubei, Hunan, Guizhou, Heilongjiang, Beijing, Shanghai, Chongqing, Shaanxi, Yunnan and Zhejiang (15) | 21422 |
